# Cell-Penetrating Ability of Peptide Hormones: Key Role of Glycosaminoglycans Clustering

**DOI:** 10.3390/ijms161126025

**Published:** 2015-11-16

**Authors:** Armelle Tchoumi Neree, Phuong Trang Nguyen, Steve Bourgault

**Affiliations:** 1 Department of Chemistry, PharmaQAM, University of Québec in Montréal, Montréal, QC H3C 3P8, Canada; tchoumi_neree.armelle@courrier.uqam.ca (A.T.N.); phuong.t.ngn@gmail.com (P.T.N.); 2 Quebec Network for Research on Protein Function, Structure and Engineering, PROTEO, Quebec City, QC G1V 0A6, Canada

**Keywords:** cell-penetrating peptides, peptide hormones, pituitary adenylate-cyclase-activating polypeptide, PACAP, secretin, glucagon, glycosaminoglycans, cellular uptake

## Abstract

Over the last two decades, the potential usage of cell-penetrating peptides (CPPs) for the intracellular delivery of various molecules has prompted the identification of novel peptidic identities. However, cytotoxic effects and unpredicted immunological responses have often limited the use of various CPP sequences in the clinic. To overcome these issues, the usage of endogenous peptides appears as an appropriate alternative approach. The hormone pituitary adenylate-cyclase-activating polypeptide (PACAP38) has been recently identified as a novel and very efficient CPP. This 38-residue polycationic peptide is a member of the secretin/glucagon/growth hormone-releasing hormone (GHRH) superfamily, with which PACAP38 shares high structural and conformational homologies. In this study, we evaluated the cell-penetrating ability of cationic peptide hormones in the context of the expression of cell surface glycosaminoglycans (GAGs). Our results indicated that among all peptides evaluated, PACAP38 was unique for its potent efficiency of cellular uptake. Interestingly, the abilities of the peptides to reach the intracellular space did not correlate with their binding affinities to sulfated GAGs, but rather to their capacity to clustered heparin *in vitro*. This study demonstrates that the uptake efficiency of a given cationic CPP does not necessarily correlate with its affinity to sulfated GAGs and that its ability to cluster GAGs should be considered for the identification of novel peptidic sequences with potent cellular penetrating properties.

## 1. Introduction

Cell-penetrating peptides (CPPs) are polypeptides generally encompassing between five and 30 residues, which can readily cross the cellular plasma membrane through different mechanisms that still remain the matter of active debates [1]. Their potent cell penetrating ability renders them as promising chemical tools for the intracellular delivery of diverse macromolecular cargoes intended for biotechnical, medical and/or diagnostics applications. However, the *in vivo* usage of several novel CPPs is often limited by their cytotoxicity and/or by the unexpected immunological responses induced upon their injection [1,2]. Thus, identification of endogenous polypeptide sequences with potent capability of transporting molecular cargoes across plasma membranes is of great interest.

CPPs are usually classified into three different classes, *i.e.*, hydrophobic, amphipathic and cationic [3]. Cationic CPPs are peptides containing several positively charged residues. The cationic peptide derived from the HIV-1 protein TAT was the first CPP identified over two decades ago [4]. Whereas numerous CPPs have been discovered through large libraries screening and from prediction models, the majority of CPPs identified so far originates from natural polypeptide sequences, including DNA/RNA-binding proteins, viral proteins, signal peptides or heparin-binding proteins [3]. It was recently observed that a peptide (neuro)hormone, pituitary adenylate-cyclase-activating polypeptide, known as PACAP, can cross the plasma membrane mainly by active transport independent of its specific membrane-bound receptor [5]. This peptide was highly effective to mediate the cellular uptake of a variety of cargoes, including large proteins and polynucleotides [6]. Interestingly, the cellular uptake efficacy of PACAP was approximately three times as high as that observed for the TAT peptide [5], highlighting the important ability of this peptidic ligand to cross cellular membrane.

PACAP is a 38-amino acid C-terminally-α-amidated peptide that was originally isolated from hypothalamic extracts according to its capacity to stimulate adenylyl cyclase from pituitary cells [7]. This peptide mediates its biological activities by specifically interacting with three types of cell surface G protein-coupled receptors (GPCRs) [8]. PACAP and its receptors are widely expressed in the central nervous system and peripheral tissues, where this peptide exerts key physiological functions [7]. Posttranslational processing of PACAP generates a 27-residue peptide (PACAP27) that exhibits similar biological activities compared to PACAP38 [7]. PACAP27 shows a high sequence identity with vasoactive intestinal polypeptide (VIP), thus identifying PACAP as a member of the secretin/glucagon/growth hormone-releasing hormone (GHRH) superfamily. Peptide hormones of this family are long and linear polypeptidic chains, which share structural and physicochemical properties [9]. These peptides exhibit primarily a random coil conformation in aqueous solutions whereas the central and C-terminal domains of the polypeptide chain readily adopt a helical structure in membrane-mimicking milieu, such as trifluoroethanol and dodecylphosphocholine (DPC) micelles [9,10,11,12]. Interestingly, members of the secretin/glucagon/GHRH superfamily display numerous basic residues dispersed throughout their central and C-terminal domains, conferring a polycationic nature to these peptides (Table 1). It was recently reported that PACAP38 is readily able to cross the plasma membrane to reach the intracellular space mainly by active mechanisms that are independent of its interaction with specific cell surface GPCRs [5]. Moreover, PACAP38 cellular uptake is dependent of the expression of glycosaminoglycans (GAGs) on the outer leaflet of the plasma membrane [13]. Nonetheless, little is known about the cell penetrating capacities of other members of this superfamily of endogenous cationic peptide hormones. Interestingly, sequences analysis and/or two dimensional projection of putative α-helix revealed that nearly all peptides of the secretin/glucagon/GHRH superfamily comprise consensus heparin-binding or Cardin-Weintraub motif [14] (XBBXBX; in which B is a basic amino acid), distributed either sequentially or spatially upon helical folding, suggestive of GAG-peptide interactions (Table 1). Thus, their polycationic nature, the presence of heparin-binding motif and the high sequence homology of these polypeptides with PACAP38 all suggest that they could also have cell penetrating ability, an avenue that has not been addressed so far.

**Table 1:** Sequence of peptides evaluated in the present study.

Peptide	Sequence ^a,b^	Charge ^c^
PACAP 38	HSDGIFTDSYS**R**Y**RK**QMAV**KK**YLAAVLG**KR**Y**K**Q**R**V**K**N**K**	10
PACAP 27	HSDGIFTDSYS**R**Y**RK**QMAV**KK**YLAAVL	4
VIP	HSDAVFTDNYT**R**L**RK**QMAV**KK**YLNSILN	4
Secretin	HSDGTFTSELS**R**LL**R**EGA**R**LQ**R**LQGLV	2
Glucagon	HSQGTFTSDYS**K**YLDS**RR**AQDFVQWLMNT	1
GLP-1	HDEFE**R**HAEGTFTSDVSSYLEGQAAQGFIAWLV**K**G**R**G	−2
Calcitonin	CGNLSTCMLGTYTQDFN**K**FHTFPQTAIGVGAP	1
TAT(48–60)	G**RKKRR**Q**RRR**PPQ	9

^a^ Basic residues arginine and lysine are indicated in bold; ^b^ All peptides are C-terminally-α-amidated; ^c^ Net charge at experimental pH 7.4.

In this context, we evaluated the uptake of peptides from the secretin/glucagon/GHRH and of calcitonin, a peptide hormone for which several fragments are known to translocate cellular membranes [15]. Our results showed that among all these peptides, PACAP38 was unique for its high cell-penetrating efficacy. Interestingly, although cell surface GAGs contributed significantly to their cellular uptakes, the efficiency of uptake did not correlate with *in vitro* binding to sulfated GAGs and to the secondary conformational conversion induced by their binding to heparin. Instead, PACAP38 was unique among these peptide hormones for its capacity to cluster sulfated GAGs *in vitro*.

## 2. Results and Discussion

### 2.1. Cell Surface Glycosaminoglycans Promote Cellular Uptake of Class B GPCR Ligands

**Figure 1:**
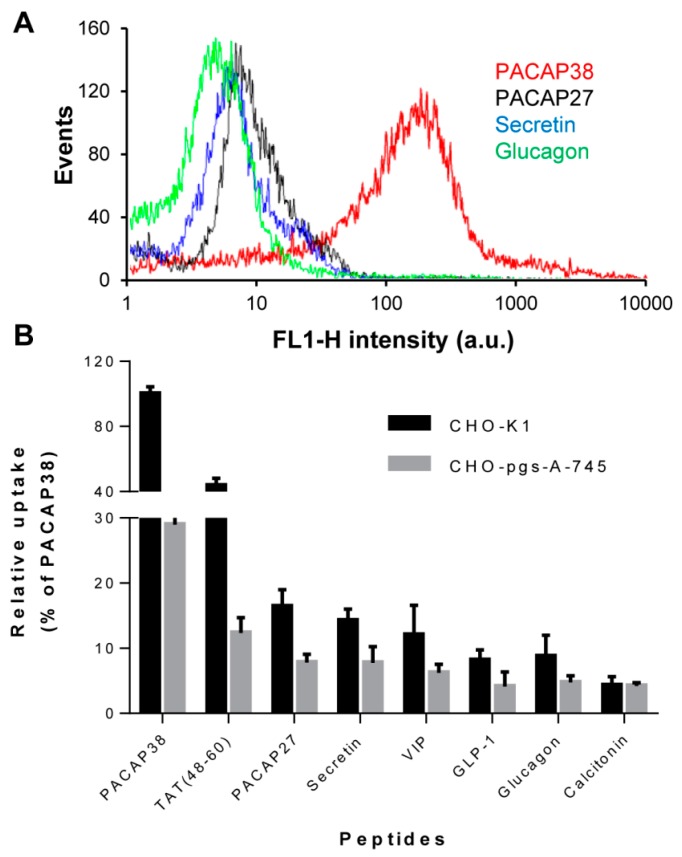
Cellular uptake of cationic peptide hormones and TAT peptide. (**A**) Representative flow cytometry histograms showing cellular uptake of 1 μM PACAP38 (red), PACAP27 (black), secretin (blue) and glucagon (green) by CHO K1 cells; (**B**) Cellular uptake of 1 μM cationic peptide hormones and TAT peptide by CHO K1 and CHO pgs-A-745 cells. Data represent the relative mean fluorescence intensity (± S.E.M.) of gated cells from at least 3 individual experiments and results are expressed as a percentage of the median fluorescence for 1 μM PACAP38 in CHO K1 cells. Cells were incubated for 1 h at 37 °C in 5% CO_2_ with fluorescently labelled peptides, washed twice in HBSS, treated with 100 μg/mL heparin for 5 min, detached by trypsin treatment, washed by centrifugation and resuspended in ice-cold sorting buffer before flow cytometry analysis.

PACAP27, VIP, secretin, glucagon and GLP-1 are members of the secretin/glucagon/GHRH peptides superfamily with whom PACAP38, a novel CPP-derivative [6], shares considerable structural and physicochemical homology (Table 1). Thus, we evaluated the cellular uptake of representative members of this peptide family and of calcitonin, a class B GPCR peptide ligand that was previously shown to efficiently translocate through the plasma membrane [16]. As observed, using flow cytometry, at 1 µM all fluorescently-labelled peptides were relatively poorly taken up by CHO-K1 cells, with internalization efficacy approximately 5- to 10-fold lower compared to PACAP38. Taken into account their primary structure (Table 1), these results are consistent with other studies that have suggested that the presence of eight positive residues is needed for the efficient internalization of cationic CPPs [17]. Nonetheless, the levels of internalization of PACAP27, VIP and secretin were around 50% of that observed for the TAT (48–60) peptide, the control CPP used in this study, indicating that these three peptides displayed some cell penetrating ability (Figure 1).

As recently reported for PACAP38 [13], the cellular uptake of representative peptides of the secretin/glucagon/GHRH peptide superfamily was partially dependent on the presence of the polysaccharide-domains of proteoglycans, as cellular uptakes were 30% to 50% lower for GAGs-deficient CHO-pgs-A-745 cells compared to their wild-type counterpart CHO-K1 cells (Figure 1B). CHO pgs-A-745 cells are deficient in xylosyltransferase, an enzyme that catalyzes the transfer of a d-xylosyl group to the side chain of a serine, a key step in the synthesis of proteoglycans. As a consequence, these cells do not express any GAGs on the outer leaflet of their plasma membrane [18]. In contrast, endocytosis of calcitonin appears to be independent of cell surface GAGs, as the extent of internalization was rather similar in K1 and pgs-A-745 CHO cells. This is in agreement with the main mechanisms of internalization proposed for calcitonin and its 9–32 fragment conferring a key role of negatively charged phospholipids in peptide cellular uptake [15]. In addition, calcitonin displays only one positively charged residue, a Lys at position 18 (Table 1), suggestive of poor putative electrostatic interactions with sulfated GAGs.

### 2.2. Relative Affinity for Sulfated Glycosaminoglycans

**Figure 2:**
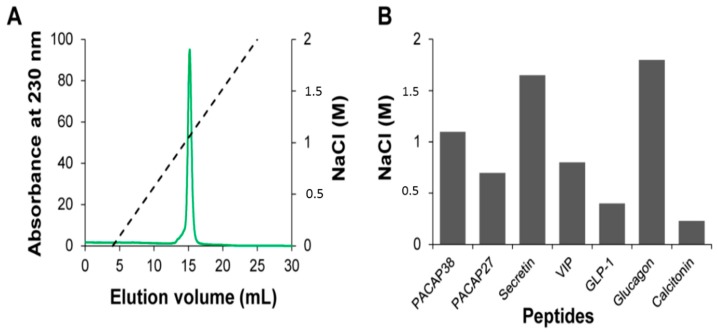
Heparin affinity chromatography of cationic peptide hormones. (**A**) Chromatograms of PACAP38 elution with increasing NaCl concentration on a sepharose heparin column connected to a FPLC Aktapure system. The NaCl gradient is shown as dashed line; (**B**) Relative affinities of peptide hormones for heparin. The histogram indicates the concentration of NaCl required for eluting a given peptide from the heparin sepharose resin; (**A**,**B**) For all experiments: injection of 250 μg of peptides in phosphate buffer (20 mM, pH 7.4) and flow rate of 0.5 mL/min.

Considering that the cellular uptakes of PACAP27, VIP, secretin, glucagon, GLP-1 and calcitonin were between 5- to 10-fold lower compared to PACAP38 and that cell surface GAGs play key roles in PACAP38 endocytosis [13], we examined if this lower extent of internalization could be the consequence of a lower binding affinity towards sulfated GAGs. To address this question, we probed the relative affinity of the peptides for heparin, an analog of the highly-sulfated domains of heparan sulfate, the most abundant GAGs on the cell surface of eukaryotic cells [19], by means of affinity chromatography. Surprisingly, despite their lower content in positive charges, secretin and glucagon showed a higher relative affinity towards heparin compared to PACAP38 (Figure 2). Indeed, secretin and glucagon display four and three basic residues, respectively, whereas PACAP38 encompasses four Arg and seven Lys. Particularly, NaCl concentrations needed to elute secretin (1.65 M) and glucagon (1.8 M), were equivalent or higher to the one reported for the poly-arginine CPPs penetratin [20] and TAT [21] and of several chemokines known to bind avidly to sulfated GAGs [19]. This data indicates that the binding mode of these peptides to heparin is not purely based on electrostatic interactions, suggesting somewhat of specificity in heparin-secretin and heparin-glucagon, an avenue that we are currently exploring. Although glucagon has only two Arg and one Lys residues, this peptide showed the higher relative affinity for heparin among all peptides studied, indicative of a unique contribution of the Arg-Arg motif located at positions 17 and 18.

While PACAP27 and VIP display a high sequence homology with secretin (Table 1), they bind relatively weakly to heparin compared to secretin. This could be related to the fact that arginine has a higher affinity for sulfated GAGs than lysine [22]. Calcitonin and GLP-1 that encompass one residue and a negative net charge at pH 7.4, respectively, bind weakly to the heparin column (Figure 2). Overall, these results indicate that the 5- to 10-fold higher cellular uptake observed for PACAP38 compared to other peptides of the secretin/glucagon/GHRH superfamily does not merely come from a higher affinity to sulfated GAGs, but most likely involves other mechanism(s). Although we showed that the cellular uptake efficacy of PACAP38 was approximately twice higher than the one observed for the TAT peptide (Figure 1B), PACAP38 eluted from the heparin-sepharose column at NaCl concentration of 1.1 M (Figure 2), whereas it has been previously shown that TAT is eluted at around 1.8 M NaCl [21]. Accordingly, the *in vitro* affinity of binding to sulfated GAGs is not an appropriate criterion to estimate the efficacy of uptake of cationic peptides.

### 2.3. Heparin Binding Induced Conformational Conversion of Peptide Hormones

**Figure 3:**
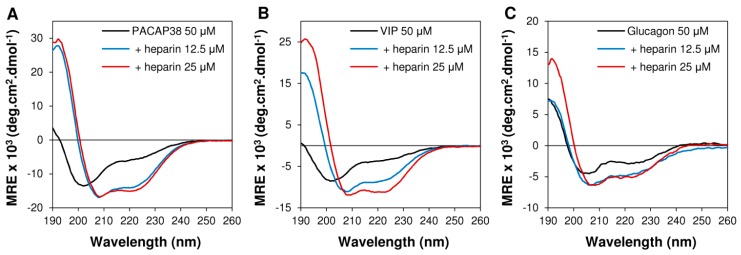
Secondary conformational conversion of peptides upon binding to heparin. Circular dichroïsm spectra of (**A**) PACAP38 (50 μM); (**B**) VIP and (**C**) glucagon (50 μM) in absence or in presence of heparin (12.5 and 25 μM). Buffer in all experiments is 20 mM phosphate, 100 mM NaF, pH 7.4 and temperature is 25 °C.

We recently reported that upon binding to sulfated GAGs, PACAP38 undergoes a random coil-to-α-helix conformational conversion [13]. Interestingly, by hindering the helical folding of PACAP38 with incorporation of d-amino acids, we observed that the GAGs-induced helical structure was essential for GAGs-dependent uptake whereas it was not critical for efficient internalization in CHO-pgs-A-745 cells [13]. Thus, we evaluated if the lower efficacy of cellular uptake of representative peptides of the secretin/glucagon/GHRH superfamily and of calcitonin could not be ascribed to the incapacity of these peptides to adopt an α-helix upon binding to GAGs. As observed by circular dichroism (CD) spectroscopy, most of the peptides used in the present study displayed a disordered structure in aqueous solution, as revealed by the presence of a single minimum between 200 and 205 nm (Figure 3: PACAP38, VIP and glucagon as representative peptides). In sharp contrast, in presence of 12.5 and 25 μM of heparin, CD spectra of VIP, PACAP38 and glucagon displayed two negative minima at 208 and 222 nm and a positive maximum at around 192 nm, indicating a major contribution of a helical conformation (Figure 3). Similar results were obtained for other members of the secretin/glucagon/GHRH superfamily. CD experiments revealed that upon binding to sulfated GAGs, all these peptides undergo a conformational conversion into a well-defined α-helix secondary structure. Moreover, it has been previously shown that upon binding to heparin, the TAT peptide, which is mostly unstructured in solution, also adopts an α-helical conformation upon its binding to heparin [23]. Thus, these data suggested that the lower extent of the cell-penetrating capacity of these peptides, in comparison to PACAP38, is not related to a lack of GAGs-induced helical folding.

### 2.4. PACAP38 Is Unique to Induce Heparin Clustering

**Figure 4:**
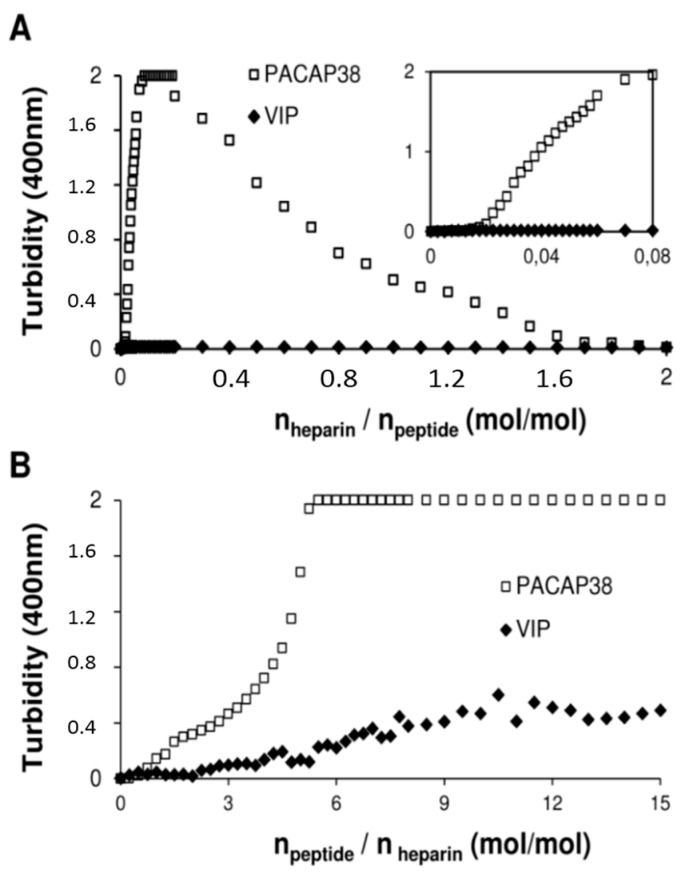
Light scattering showing heparin clustering by PACAP38 and not by other peptides. (**A**) Titration of heparin into PACAP38 (□) or VIP (◆) monitored by turbidity at 400 nm. Each peak corresponds to the injection of 1.25 μL of a 100 μM heparin solution (20 mM phosphate, 100 mM NaCl, pH 7.4) into a 1 mL 50 μM peptide solution (20 mM phosphate, 100 mM NaCl, pH 7.4). Inset: emphasis on the initial phase of the titration assay; (**B**) Titration of PACAP38 (□) and VIP (◆) into heparin monitored by turbidity at 400 nm. Each peak corresponds to the injection of 12.5 μL of a 200 μM peptide solution (20 mM phosphate, 100 mM NaCl, pH 7.4) into a 1 mL 10 μM heparin solution (20 mM phosphate, 100 mM NaCl, pH 7.4). Data are expressed as a function of (**A**) heparin/peptide molar ratio or (**B**) peptide/heparin molar ratio.

It has been previously shown that GAGs clustering plays a pivotal role in the endocytosis of cationic CPPs, such as WR9 [24] and penetratin [20]. We already reported that when PACAP38 is titrated into a heparin solution, the solution becomes rapidly turbid [13]. Therefore, we investigated the capacity of these peptides to cluster heparin in order to elucidate the molecular basis of the unique GAGs-dependent cell penetrating capacity of PACAP38 among the VIP/secretin/GHRH superfamily. We analyzed the formation of molecular heparin-peptide complexes by monitoring the increase of solution turbidity at 400 nm upon the titration of each peptide into heparin. When heparin (100 μM) was successively titrated into PACAP38 solution (50 μM), we observed a rapid increase of turbidity after an initially baseline during the first few injections (Figure 4A). When increasing the heparin/peptide ratio, the solution became less turbid at a molar ratio of 0.8 and higher. In sharp contrast, titration of heparin (100 μM) into a VIP (50 μM) solution did not lead to any increase of turbidity (Figure 4A). This absence of turbidity at 400 nm in the heparin-into-peptide titration was also observed for PACAP27, secretin, glucagon, GLP-1 and calcitonin (data not shown), indicating that PACAP38 is unique among peptides used in this study for inducing the formation of large particles upon heparin binding that scatter light at 400 nm. Similarly, in the peptide-into-heparin titration experiment, PACAP38 solution showed a significant increase of turbidity at a molar ratio of *n*_peptide/heparin_ = 2 and higher (Figure 4B). In contrast, VIP, as well as the other peptides used in the present study displayed a weak light scattering signal in the peptide-into-heparin titration, with a slight increase of turbidity at a molar ratio of 6 and higher (*n*_peptide/heparin_) (Figure 4B). This turbidity titration experiment suggested that the high cellular uptake efficacy of PACAP38, in contrast to representative peptides of the secretin/glucagon/GHRH family and of calcitonin, could be related to the high capacity of PACAP38 to form macromolecular clusters with heparin *in vitro*.

## 3. Experimental Section

### 3.1. Peptide Synthesis, Purification and Characterization

All peptides were synthetized on a Tribute peptide synthesizer (Protein Technologies, Tucson, AZ, USA) with standard Fmoc chemistry using 2-(6-Chloro-1-H-benzotriazole-1-yl)-1,1,3,3-tetramethylaminium hexafluoro-phosphate (HCTU) as a coupling reagent and diisopropylethylamine (DIEA) as a base. For fluorescein labeling, Fmoc-Ahx-OH was first coupled at the N-terminus of each peptidyl-resin. After Fmoc removal, a solution containing fluorescein isothiocyanate (FITC; 3 eq.), triethylamine (TEA; 3 eq.) in DCM:DMF (1:1) was added to the reaction vessel and was allowed to react overnight at room temperature. FITC coupling was monitored using the ninhydrin test. Peptides were cleaved from the resin using a mixture of TFA:ethanedithiol:phenol:water (92:2.5:3:2.5; *v*/*v*). After filtration and evaporation of the cleavage mixture, peptides were precipitated and washed with diethylether, solubilized in water and lyophilized. Crude peptides were purified by reverse-phase high performance liquid chromatography (RP-HPLC) on a preparative Luna C_18_ column (250 mm × 21.2 mm; 5 µm, 100 Å, Phenomenex, Torrance, CA, USA) using a linear gradient of ACN in H_2_O/TFA (0.06% *v*/*v*). Collected fractions were analyzed by analytical RP-HPLC using an Aeris peptide XB C_18_ column (150 mm × 4.6 mm; 3.6 µm, Phenomenex) and by ESI-TOF mass spectrometry. Fractions corresponding to the desired peptides, as confirmed by mass spectrometry, with purity higher than 95%, measured by analytical HPLC, were finally pooled and lyophilized.

### 3.2. Cell Culture

Chinese hamster ovary cells K1 (CHO K1; obtained from ATCC, Manassas, VA, USA) and xylosyltransferase deficient cells [18] (CHO pgs-A-745; obtained from ATCC) were maintained in Ham’s F12 medium supplemented with 10% foetal bovine serum (FBS), 2 mM l-glutamine and antibiotic (10,000 UI/mL penicillin, and 10,000 UI/mL streptomycin). Cells were maintained as a monolayer at 37 °C in a humidified atmosphere of 5% CO_2_ and 95% air and passaged by trypsinization when the cells reached 70%–80% confluence.

### 3.3. Evaluation of Peptide Uptake by Flow Cytometry

For cellular uptake measurement, CHO K1 and CHO pgs-A-745 were seeded in 12-well plates at a density of 30,000 cells/well for 48 h to approximately 75% confluence. After removing the culture media, cells were incubated in 1% FBS Ham’s F12 medium (supplemented with glutamine and antibiotic/antimycotic) in presence of different concentrations of fluorescently labelled peptide for 1 h at 37 °C and 5% CO_2_. The time of incubation was defined according to our previous study showing that the maximum uptake was obtained upon 1 h incubation [5]. After incubation, cells were washed twice with HBSS buffer, treated for 5 min at room temperature with 100 μg/mL heparin in HBSS to remove the excess of peptide bound to the cell surface [25,26], washed once again with HBSS and detached by trypsinization (5 min) at 37 °C. Trypsin action was stopped by the addition of complete Ham’s F12 media supplemented with 10% FBS and cells were centrifuged for 5 min at 400× *g*. Cells were resuspended in 500 μL ice-cold sorting buffer (Ca/Mg free PBS, 1 mM EDTA, 25 mM HEPES, 1% FBS) and kept on ice until flow cytometry analysis. To confirm that the fluorescence measured by flow cytometry was not associated to non-specific binding of the FITC-peptides at the cell surface, we performed the analysis after treatment with the fluorescent quencher trypan blue. Cells that were incubated with FITC-PACAP38, were treated with 0.25 mg/mL of trypan blue for 1 min immediately before flow cytometry analysis. This treatment did not reduced significantly the level of the fluorescence measured (data not shown), suggesting that trypsin and heparin treatments were sufficient to remove the peptide absorbed non-specifically to the plasma membrane.

### 3.4. Affinity Chromatography

The relative binding affinity of the peptides for sulfated GAGs was evaluated using a 1 mL HiTrap Heparin HP column packed with sepharose and immobilized heparin from porcine intestinal mucosa (GE Healthcare, Baie-d'Urfé, QC, Canada). The column was connected to a FPLC Aktapure system (GE Healthcare) and equilibrated with phosphate buffer (20 mM, pH 7.4) prior to injection. Five hundred microliters of 0.5 mg/mL peptide solutions were injected at a flow rate of 0.5 mL/min. After injection, the elution consisted of an isocratic step of 5 mL phosphate buffer (20 mM, pH 7.4) followed by a gradient from 0 to 2 M NaCl in phosphate buffer (pH 7.4). Peptide elution was monitored using absorbance at 230 nm.

### 3.5. Circular Dichroism Spectroscopy

Far-ultraviolet circular dichroism (CD) spectra were recorded at room temperature using a Jasco J-810 CD spectrometer (Jasco, Easton, MD, USA). Peptide solutions, without or with increasing concentrations of heparin (from porcine intestinal mucosa) were prepared in phosphate buffer (20 mM, 100 mM·NaF, pH 7.4). All spectra were measured from 260 to 190 nm and were corrected by subtracting the appropriate blank solution (with or without heparin). A 1 mm path length quartz cuvette was used.

### 3.6. Clustering Measured by Turbidity

The formation of macromolecular GAG-peptide clusters was monitored by measuring the light scattering at 400 nm, as previously described [27,28], using a 100 Bio Cary UV-visible spectrophotometer (Varian, Malton, ON, Canada). Briefly, for the heparin-into-peptide titration, a 1 cm length quartz cuvette was filled with 1 mL of a 50 μM peptide solution (20 mM phosphate buffer, 100 mM·NaCl, pH 7.4) and 1.25 μL aliquots of a 100 μM heparin solution (20 mM phosphate buffer, 100 mM·NaCl, pH 7.4) were successively added. The solution was gently mixed by vortex and the turbidity at 400 nm was immediately measured. For the peptide-into-heparin titration, a 1 cm length quartz cuvette was filled with 1 mL of a 10 μM heparin solution (20 mM phosphate buffer, 100 mM·NaCl, pH 7.4) and 12.5 μL aliquots of a 200 μM peptide solution (20 mM phosphate buffer, 100 mM·NaCl, pH 7.4) were successively added. The solution was gently mixed by vortex and the turbidity at 400 nm was immediately measured. Data was expressed as solution turbidity at 400 nm as function of peptide/heparin or heparin/peptide molar ratio.

## 4. Conclusions

It has been previously shown that the (neuro) hormone PACAP38 and several of its derivatives are efficiently competent to cross the plasma membrane and to deliver a variety of cargoes inside the cell [5,6]. Additionally, as previously observed for other cationic CPPs, we recently reported that the uptake efficacy of PACAP38 is dependent on the expression of cell surface GAGs [13]. The high sequence and structural homology of PACAP38 with peptides of the secretin/glucagon/GHRH superfamily suggest that these cationic peptidic hormones could have cell-penetrating capacity.

Evaluation of the cellular uptake of these peptide hormones revealed that these polypeptides were poorly internalized in comparison to PACAP38. Nonetheless, their cell uptake efficacies were dependent on the presence of cell surface GAGs. Interestingly, although secretin and glucagon bind to the immobilized heparin with a higher relative affinity compared to PACAP38, they were poorly internalized by CHO K1 cells. This data demonstrates that there is no direct relationship between the *in vitro* binding to sulfated GAGs and the cellular uptake efficacy of potential cationic CPPs, as previously revealed for hLF peptide derivatives [29]. Instead, we observed that among all peptides tested in the present study, PACAP38 was unique for its very high ability to form large macromolecular aggregates that scatter light at 400 nm upon its titration into a heparin solution. This observation strongly suggests that the distinctive GAGs-dependent endocytosis of PACAP38 among peptides of the secretin/glucagon/GHRH family correlates with its strong capacity to cluster heparin *in vitro*. Moreover, this study demonstrates that the uptake efficiency of a given cationic CPP does not necessarily correlate with its *in vitro* binding to sulfated GAGs. Instead, the ability to cluster GAGs *in vitro* should be taken into account for the identification of novel peptidic identities with potent cellular penetrating properties.
